# Leaving the labour market later in life: how does it impact on mechanisms for health?

**DOI:** 10.1136/oemed-2016-104258

**Published:** 2017-08-21

**Authors:** Elise Whitley, Frank Popham

**Affiliations:** MRC/CSO Social and Public Health Sciences Unit, University of Glasgow, Glasgow, UK

**Keywords:** epidemiology, ageing, disease, disease type, Retired, speciality

## Abstract

**Objectives:**

Negative associations between non-employment and health among older people are well established and are potentially important for successful ageing. However, opportunities to improve health through re-employment or extending working lives are limited as later-life exits from employment are often unwanted and permanent. We aim to establish a greater understanding of the psychosocial mechanisms underlying non-employment and health associations in older people to identify modifiable pathways through which the negative impact of non-employment can be ameliorated.

**Methods:**

Using multilevel analysis of four waves of repeated panel data from a representative sample of 1551 older men and women reaching state retirement age in the West of Scotland from 1987/1988 to 2000/2004, we explored respondents' strength of agreement with 20 statements relating to their self-defined employment status, covering themes of functioning, social engagement, self-esteem, mental engagement, stress, and control and autonomy.

**Results:**

Compared with those in employment, respondents who were retired, unemployed, sick/disabled and home makers were more likely to agree that this resulted in poor social engagement, low self-esteem and, with the possible exception of retirees, reduced mental engagement. Associations were particularly marked among unemployed and sick/disabled respondents who also agreed that their status was a source of worry and prevented them from feeling in control.

**Conclusion:**

Older people who are not in employment are at higher risk of poor physical and mental health. Interventions targeting psychosocial mechanisms such as social and mental engagement and self-esteem offer potentially valuable opportunities to improve health outcomes and promote successful ageing.

**What this paper adds**

- Non-employment is associated with poor health outcomes, but opportunities to intervene via re-employment or extending working lives is not always possible, particularly among older people for whom workplace exits are often unwanted and permanent.
- A good understanding of the psychosocial mechanisms underlying associations between non-employment and health may provide opportunities to improve health outcomes, but empirical studies of this type are rare, do not control for unobserved confounding and are often limited to single employment or non-employment states.
- Based on multilevel analysis of representative panel data, older individuals who were retired, unemployed, sick/disabled and home makers were more likely than those in employment to agree that their status had a negative impact on social and mental engagement and on their self-esteem.
- Associations were particularly strong in unemployed and sick/disabled individuals who also felt that their status was a source of worry and prevented them from feeling in control.
- Although re-employment may not always be possible, interventions that decrease loneliness and social isolation and improve self-esteem and mental engagement may ameliorate the negative impact of non-employment in older people.

## Introduction

It is well established that health and labour market status are associated, with worse health recorded for those of working age who are classified as unemployed, sick or disabled.^1 2^ While poor health itself may be a reason for non-employment, it is also broadly accepted that the converse is true, namely that labour market status can affect health.^3^ Moreover, variation in employment rates over time^4^ and internationally^5^ demonstrates that those with disabilities or poor health can be successfully employed and that being employed may have benefits for health^6^ and longevity.^7^ These findings are particularly pertinent in the context of recent and continuing worldwide increases in longevity and improved health among older individuals.^8^ These population trends have led to a rapid interest in the idea of ‘successful ageing’, with growing recognition of the importance of maintaining good physical and mental health and also of continuing social, productive and economic activity in later life.^9 10^ Successful ageing is an important goal for both individuals and society as continuing participation in the workforce by older people has the potential to increase economic activity and reduce healthcare and pension costs. As a result, many countries are developing policies aimed at extending working lives.^8^ However, many individuals in Organisation for Economic Co-operation and Development (OECD) countries effectively retire before their state’s pension age,^11 12^ although this may now be changing, with the proportion of individuals aged 55–64 years participating in the workforce slowly increasing from the 2000s onwards.^12^

In addition to the timing of exit from employment, it is also important to consider the route taken.^2 12^ Reasons for leaving the workplace vary according to factors such as age, gender and socioeconomic position, and these different routes are likely to have distinct impacts on health. Specific to older workers, and in contrast to unemployment, retirement can have positive benefits for health, particularly mental health, with greater benefits for those retiring from jobs with less than ideal working conditions.^13–15^ However, the health impacts of formal retirement may be different to *de facto* retirement associated with job loss and classification as unemployed or sick or disabled. A recent European report found that over 30% of retirees would have preferred to keep working past the time they retired, with ‘push factors’ such as job loss and ill health driving early retirement rather than ‘pull factors’ such as incentive schemes.^16^ Data from OECD countries reveal the scale of this problem, with ‘at least half of men using routes such as unemployment, sickness or disability benefits in half of countries’, while many women leave the job market early to care for family members.^12^ Socioeconomic inequalities in retirement route are stark,^11^ with the most advantaged able to retire securely, often early, with occupational or private pensions, while the most disadvantaged are more likely to exit early via unemployment and disability, or have to work to state pension age as they lack the means to retire early. This is an increasingly important distinction as recent decades have seen rises in OECD countries of older disadvantaged workers not in employment and receiving long-term unemployment or sickness/disability benefits with little hope of returning to work.^17–19^

The impact of non-employment on health is a particular issue in the context of ageing as exits from the workplace among older individuals are often permanent^20^ and opportunities to improve health through return to employment or extension of working life may be limited. However, a greater understanding of how and why labour market status impacts on health, that is, identifying the mechanisms underlying these associations, provides an opportunity to ameliorate the negative impact of non-employment in older people by tackling these potentially modifiable pathways. In addition to economic benefits, employment provides important psychosocial functions, including social contact, daily structure, collective purpose, social identity, status and regular activity,^21^ all with possible consequences for health. For example, loneliness and social isolation (two distinct notions) are well recognised problems among older individuals^22^ and have been shown to be associated with worse mental and physical health, acting through factors such as poor health behaviours, anxiety, poor sleep, fatigue and cognitive decline.^23–25^ Stress due to unemployment^1^ is a well-known risk factor for poor health while, in contrast, retirement from a high stress job may result in an improvement in health,^13 26^ highlighting the importance of considering distinct impacts of different exits from the workplace. Low levels of variation, control or autonomy at work are associated with greater morbidity,^27 28^ and lack of control around retirement may also adversely affect health.^29^ In addition, societal attitudes towards the non-employed may have a negative psychological impact, particularly among those who face stigma because of poor health.^30 31^

However, in spite of the potential importance, well-controlled empirical studies of the mechanisms underlying associations between employment status and health outcomes are rare, even in the case of the most extensively studied labour force states. For example, a recent systematic review of longitudinal studies of retirement and health highlights the need for further research into potentially influencing factors,^32^ while a meta-analysis of the relationship between unemployment and mental health specifically reports a dearth of mechanism studies.^33^ Much of the evidence to date is based on comparison of employed individuals with a single non-working group, for example, the unemployed^1 34 35^ or retired,^13 26^ or on the exploration of potential mechanisms among individuals in a single state, for example, unemployed^36^ or employed, using occupational cohorts,^27 28^ often exclusively male. There is therefore a lack of evidence comparing multiple exit routes from the workplace in the general population, including women. In addition, information on mechanisms of interest is often based on general questions such as: ‘Are you currently physically/mentally tired?’^14^ or ‘Have you been feeling tense, stressed, or under a lot of pressure during the past month?’^35^ rather than assessing their direct relevance to the respondent’s current employment status.

We have explored potential mechanisms linking employment status and health using panel data from a representative sample of male and female workers reaching state retirement age in the deindustrialising context of the West of Scotland from 1987/1988 to 2000/2004, exploiting a series of questions directly accessing respondents’ feelings towards their employment status and using methods that control for time-invariant unobserved confounding. Our aims in these analyses were: (1) to robustly identify mechanisms associated with non-employment that could be important for health (functioning, social engagement, self-esteem, mental engagement, control and autonomy, and stress) and (2) to explore whether the associations vary according to the specific route taken out of the workplace (retirement, unemployment, sickness/disability and home making).

## Methods

The West of Scotland Twenty-07 study is a population-based multiple-cohort study and has previously been described in detail.^37^ In brief, it consists of three age-cohorts of men and women born around 1932, 1952 and 1972 and followed for 20 years. Each cohort consists of two samples: the first (the regional sample) is a two-stage stratified random sample of people living in an area of the West of Scotland centred on Glasgow previously known as the Central Clydeside Conurbation, and the second (the locality sample) is a sample of residents from two areas of the city of Glasgow. Respondents have been shown to be representative of the population of the sampled area. Baseline interviews were conducted in 1987/1988 (wave 1), when the three cohorts were aged approximately 55, 35 and 15 years. There were four follow-up waves in 1990/1992 (wave 2), 1995/1997 (wave 3), 2000/2004 (wave 4) and 2007/2008 (wave 5), and ethics approval was gained for each wave from the National Health Service and/or Glasgow University Ethics Committees. Current analyses are based on the oldest (1932) cohort using data from waves 1–4, when questions on feelings towards employment status were included. These respondents were aged around 55 years at wave 1 and around 70 years at wave 4 and follow-up therefore comfortably spans a 15-year period around usual retirement ages at the time (65 for men and 60 for women).

At each wave, respondents were asked whether they would describe their current employment status as: employed/worker/self-employed, retired, disabled/invalid/permanently sick, unemployed or caring for home/housewife. Respondents were also asked to respond to statements specifically relating to their current employment status. No reference period was specified for these statements, and they therefore reflect respondents’ general feelings towards their status rather than focusing on a single period. The statements are listed in table 1 with (Status) replaced by (Being retired), (Being unable to work because of illness or disability), (Caring for home), (Being unemployed) or (Being employed) as appropriate, for example: ‘Being retired is bad for my health’. In each case, respondents were asked if the statement applied: never, only occasionally, quite frequently or very frequently. For the current analyses, statements were grouped into six broad themes: functioning, social engagement, self-esteem, mental engagement, control and autonomy, and stress. However, in the interviews, statements were presented in an apparently random order that did not imply any underlying structure or themes (http://2007study.sphsu.mrc.ac.uk/Interview-Schedules.html). As shown, 11 statements were included in wave 1, while an additional nine were also included in waves 2, 3 and 4. Statements were presented to all respondents at all waves with the exception of those who were sick/disabled in wave 1 and those in the locality sample in wave 3. The majority of statements regarding employment status were negative, and responses to these were coded from 1 (never) to 4 (very frequently). For consistency of interpretation, those that could be regarded as potentially positive were coded in reverse from 1 (very frequently) to 4 (never).

**Table 1:** Statements regarding current employment status

	Included in waves	Reverse coded	Number of responses
Functioning			
(Status) is bad for my health	2, 3, 4		2547
(Status) leaves me physically tired at the end of the day	2, 3, 4		2580
(Status) leaves mentally tired at the end of the day	1, 2, 3, 4		3917
Social engagement			
(Status) can be quite lonely	2, 3, 4		2582
(Status) makes me feel isolated	1, 2, 3, 4		3923
(Status) allows me to be sociable and meet people	1, 2, 3, 4	√	3923
Self-esteem			
(Status) lets me make full use of my abilities	1, 2, 3, 4	√	3905
(Status) lets me feel important and worthwhile	1, 2, 3, 4	√	3873
Mental engagement			
(Status) is boring	1, 2, 3, 4		3925
(Status) is interesting and challenging	1, 2, 3, 4	√	3874
(Status) is too routine	2, 3, 4		2576
(Status) requires me to concentrate hard	2, 3, 4		2578
Stress			
(Status) is full of stress	1, 2, 3, 4		3924
(Status) causes me a lot of worry	1, 2, 3, 4		3925
(Status) is more than I can cope with	2, 3, 4		2578
(Status) is too frantic and hurried	2, 3, 4		2579
Control and autonomy			
(Status) prevents me feeling in control of things	2, 3, 4		2566
(Status) forces me to do what other people want	2, 3, 4		2570
(Status) leaves me plenty of time for myself	1, 2, 3, 4	√	3917
(Status) allows me to set my own pace of life	1, 2, 3, 4	√	3924

Our data are hierarchal with respondents as the higher level unit and time (survey waves 1–4) as the lower level unit, nested within respondents. We therefore used a multilevel modelling approach, recognising that observations from the same respondent are likely to be correlated, by including a person-level random effect. This approach is preferable to standard regression that ignores this interdependence. In our dataset, there are two sources of variation: between-person variation and within-person variation. A standard multilevel random effects model uses both sources of variation to calculate a single effect estimate by pooling between-person and within-person estimates. However, many researchers prefer to focus just on the within-person estimates as these control for confounding by respondents’ fixed characteristics (eg, personality).^38^ We therefore used a model that produced both between-person and within-person estimates by simultaneously including the average proportion of survey waves respondents spent in each employment state (between-person estimate) and the deviation of each individual survey wave employment state from the respondent average (within-person estimate).^39^ In this model, the between-person estimates are based on comparison of an individual with a particular employment status versus other individuals with a different employment status. In contrast, the within-person estimates are based on comparison of survey waves in a particular employment state versus waves in a different state *for the same person*. By effectively using an individual as their own control, other factors are implicitly fixed and within-person associations, which control for time-invariant personal characteristics, are therefore more robust. Separate models were fitted for each statement and were adjusted for age and sex. Analyses were based on least squares regression comparing retirement, sickness/disability, unemployment and home making with employment. All analyses were carried out using Stata V.13.0. For simplicity, we present within-person associations. In this context, positive differences indicate greater agreement with negative statements (or disagreement with positive statements) in *survey waves* in which respondents were retired, sick/disabled, unemployed or home makers when compared with *waves* in which they were employed. Between-person associations, comparing differences between individuals, were similar although somewhat more marked, potentially due to uncontrolled confounding. These are presented in supplementary materials (see online supplementary 1).

10.1136/oemed-2016-104258.supp1 Supplementary material 1

In sensitivity analyses, separate analyses were performed for those whose main lifetime occupation was manual versus non-manual and for men versus women and results were generally consistent across subgroups. Additionally, analyses were repeated excluding respondents who had held their current employment status for a year or less and results were almost identical to those presented here.

## Results

The original (wave 1) cohort consisted of 1551 individuals. Of these, 1487 (96%) were alive at wave 2 and 1259 (85%) were interviewed. In wave 3, 1386 (89%) were alive and 1030 (74%) interviewed and in wave 4, 1242 (80%) were alive and 838 (67%) were interviewed. Among those who were alive at each wave, those who were interviewed were broadly similar to those who were not in terms of age and gender. However, those not interviewed were somewhat more likely to have been sick/disabled or unemployed in the previous wave. Less than 1% of those interviewed at each wave had a missing employment status and a similar proportion had missing values for the majority of statements regarding feelings about employment, with just one or two statements having missing values for 2% or 3% of respondents. The number of responses given to the statements across all four waves is given in table 1; statements presented at all four waves attracted around 3900 responses in total, while those presented at waves 2–4 resulted in approximately 2500 responses.

**Figure 1:**
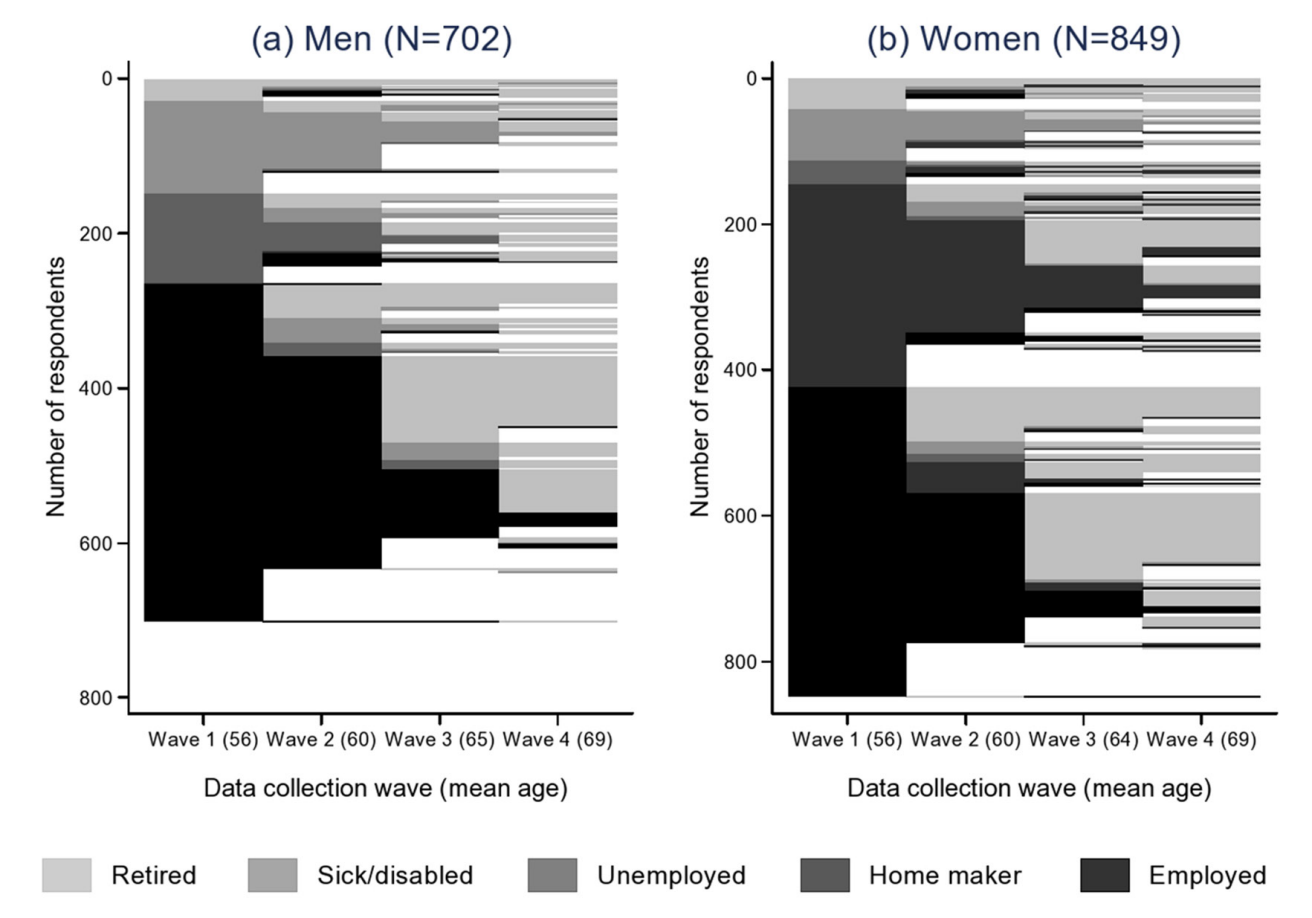
Employment status and mean age of men and women at waves 1, 2, 3 and 4.

Figure 1 presents the employment biographies of respondents across the four waves, along with their average age at each. The figure is composed of horizontal lines, each representing employment transitions between waves for an individual. Changes in colour represent changes in employment status as shown in the legend and changes to white indicate missing employment status or censoring due to death or non-participation. Figure 1a, based on 702 men, shows that in wave 1 (mean age 56 years) around 62% were in employment compared with 17% sick/disabled, 17% unemployed, and less than 5% retired. Employment status changed for some men between waves 1 and 2 (mean age 60), most commonly those who were employed or unemployed in wave 1, but 56% retained the same status. There were more marked changes to status between waves 2 and 3 (mean age 65) with 52% of men who took part in the wave stating that they were retired, and this proportion rose further to 90% in wave 4 (mean age 69), reflecting usual retirement ages at the time. Similar patterns are evident in Figure 1b, based on 849 women, with 50% of women employed and a further 33% identifying as home makers in wave 1 (mean age 56). Again there were changes in status moving to wave 2 (mean age 60), most commonly to retirement, but many women kept the same status. The most marked change observed in women’s status was between waves 2 and 3 (mean age 64) with 64% retired by this time. By wave 4 (mean age 69) 83% of women were retired and 10% considered themselves to be home-makers. Among those who changed their status between waves, the mean (SD) duration of the new status was 3.4 (1.1) years.

**Figure 2:**
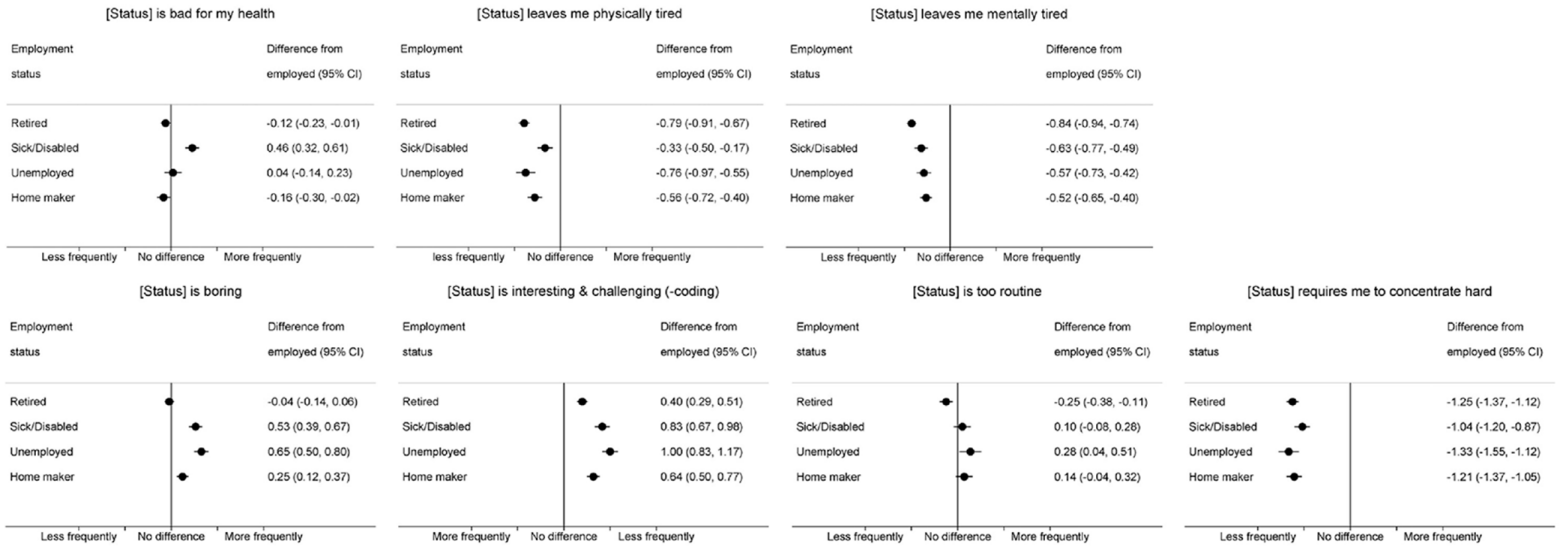
Within-person differences in feelings about employment status (functioning and mental engagement) comparing non-employed versus employed waves.

Within-person differences in responses to statements about functioning and mental engagement, comparing waves in which individuals were non-employed with those in which they were employed, are presented in figure 2. In each case, the vertical line represents no difference; points to the right of this line are generally consistent with more negative feelings in non-employed waves and points to the left with more positive feelings. Respondents in sick/disabled waves were more likely to agree that this was bad for their health (difference (95% CI): 0.46 (0.32 to 0.61)), while unemployed and employed waves were very similar. In contrast, respondents in retired and home-making waves were marginally less likely to agree that their status was harmful (−0.12 (−0.23, −0.01) and −0.16 (−0.30, −0.02), respectively). Respondents in all non-employed waves were less likely to agree that their status left them physically or mentally tired (eg, retired: −0.79 (−0.91, −0.67) and −0.84 (−0.94, −0.74), respectively). Respondents in home-making, sick/disabled and unemployed waves were considerably more likely to report that their status was boring and, along with retired waves, were less likely to find their status interesting and challenging. Again, these effects were strongest for sick/disabled (eg, boring: 0.53 (0.39, 0.67)) and unemployed (0.65 (0.50, 0.80)) waves. In terms of finding their status too routine, there was greater agreement in sick/disabled waves (0.28 (0.04, 0.51)), and less in retired waves (−0.25 (−0.38, −0.11)). Respondents in non-employed waves, regardless of the specific status, were markedly less likely to agree that their status required them to concentrate hard.

**Figure 3:**
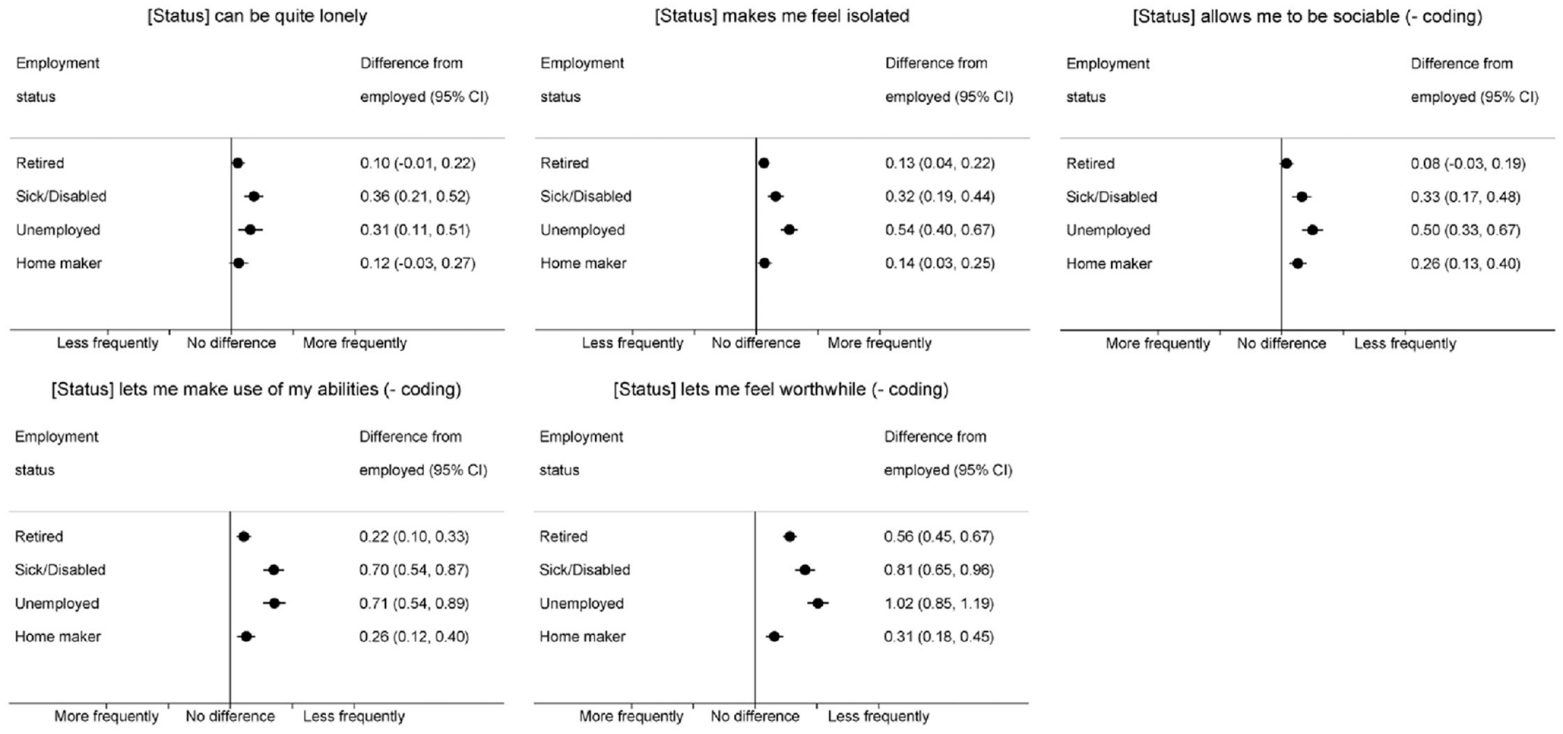
Within-person differences in feelings about employment status (social engagement and self-esteem) comparing non-employed versus employed waves.

Respondents in non-employed waves were more negative about the impact of their status on all aspects of social engagement and self-esteem (figure 3), reporting greater frequencies of feeling lonely and isolated, and lower frequencies of being sociable, making use of their abilities and feeling worthwhile. Differences were most marked in sick/disabled and unemployed waves (eg, loneliness: 0.36 (0.21, 0.52) and (0.31 (0.11, 0.51); (not) making use of abilities: 0.70 (0.54, 0.87) and (0.71 (0.54, 0.89), respectively).

**Figure 4:**
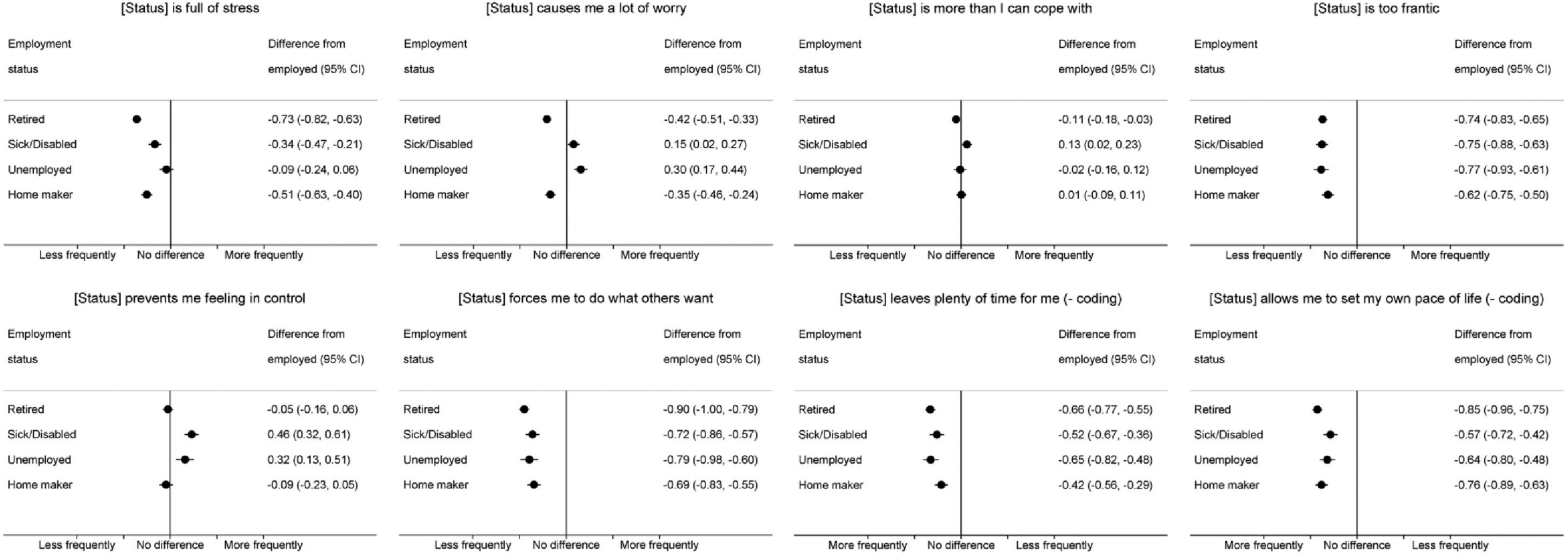
Within-person differences in feelings about employment status (stress and control and autonomy) comparing non-employed versus employed waves.

Respondents in retired (−0.73 (−0.82, −0.63), sick/disabled (−0.34 (−0.47, −0.21)) and home-making waves (−0.51 (0.63, −0.40)) were less likely than in employed waves to find their status stressful (figure 4). Those in retired and home-making waves were also less likely to agree that this status caused them worry, while those in sick/disabled and unemployed waves agreed more strongly with this statement (0.15 (0.02, 0.27) and (0.30 (0.17, 0.44), respectively). Differences between employed and non-employed waves in terms of being able to cope were small, with slightly less agreement among retired (−0.11 (−0.18, −0.03)) and slightly more among sick/disabled (0.13 (0.02, 0.23)) waves. Respondents in non-employed waves were consistently less likely to report that their status was too frantic. In terms of feeling in control, responses in retired, home-making and employed waves were very similar, while those in sick/disabled and unemployed waves were more negative (0.46 (0.32, 0.61) and 0.32 (0.13, 0.51) respectively). However, in terms of being forced to do what others want, having time for themselves and being able to set their own pace of life, respondents in non-employed waves, regardless of the reason, gave more positive responses than in employed waves.

## Discussion

We have explored potential mechanisms underlying associations between employment status and health using data from a large representative cohort of men and women who were followed up over 15 years from ages 55 to 70 years, a period comfortably spanning usual retirement ages at the time. We considered responses to statements directly asking respondents about their feelings towards their current employment status and compared employed respondents with those in four distinct non-employment states using a multilevel modelling approach that offers more robust (within-person) measures of effect. However, there are also a number of limitations that should be considered. Although our cohort was large, there was inevitably some attrition during follow-up, and all questions were not asked of all respondents at all waves, introducing the possibility of bias or measurement error due to missing values, particularly among sick/disabled or unemployed respondents. The statements on attitudes to employment status covered a wide range of potential mechanisms, many consistent with proposed psychosocial frameworks.^1 21^ However, they were not exhaustive and, in particular, we lack direct data on respondents’ perceptions of the impact of their employment status on health behaviours and financial status. In addition, some statements make less sense in the context of particular employment states, for example, ‘Being unable to work because of illness or disability is bad for my health’. At each wave, respondents classified themselves as employed, retired, sick/disabled, unemployed or home maker. While aggregation of this type is common in the literature and necessary to maintain statistical power, individual circumstances within an employment state will vary, with potentially differential impacts on health, for example, retiring from employment with favourable versus adverse working conditions.^13–15^ However, the present analyses give an indication of attitudes towards different exits from the workplace in general, and it is of note that results for those leaving manual and non-manual occupations were similar. In addition, the aggregation will include employment states of differing duration and at different ages. The impact of leaving employment changes over time^13^ and attitudes towards non-employed states after many years may not be due specifically to exiting the work place. However, the within-person associations presented here are based on recent changes in status between waves, typically of around 3 years, and attitudes are therefore likely to be relevant to respondents’ newly changed circumstances. Finally, the current analyses is based on individuals aged 55–70 years and, although it is plausible that results for sick/disabled, unemployed and home makers may extend to younger age groups, it is not possible to explicitly demonstrate it in these data. In addition, the impact of leaving employment ‘early’ at age 55 years may be different to leaving at age 65 or 70 years when the majority of individuals are retired, although associations presented here are adjusted for age, which goes some way to addressing the issue.

Previous work has reported that non-employed individuals have worse physical and mental health than those in employment,^1 2^ although evidence also suggests that retirement may have a positive impact on health.^13–15^ Our results are consistent with these views: compared with employed waves, respondents in sick/disabled waves were more likely to agree that this was bad for their health while those in home-making and retired waves took the opposite view. However, respondents in all non-employed waves, regardless of the reason, were less likely to agree that their status left them physically or mentally tired. The health implications of this finding are unclear: lower tiredness levels might indicate improved vitality in the non-employed arising from fewer of the physical and cognitive demands associated with working or, equally, might reflect a more sedentary lifestyle among those not working, with negative implications for health. Consistent with this, our results regarding mental engagement suggest that, with the possible exception of retirees, respondents in non-employed waves were more likely than in employed waves to consider their status boring and routine, potentially missing the stimulation and structure of the workplace.

Some of the most consistent associations observed in the current analyses were those relating to social engagement and self-esteem, with older individuals in non-employed waves feeling more negative about their status across both dimensions, particularly in unemployed and sick/disabled waves. These results support the notion that, in addition to financial rewards, employment provides individuals with a sense of belonging and value to society, which diminishes after leaving the workplace even when this exit is through a potentially positive route such as retirement. Loneliness, driven by both social isolation and low self-esteem, is reported as a growing problem among older people, with estimates in the UK suggesting that the prevalence among those aged 65+ is ‘clustered around the 30%–35% mark’.^22^ Loneliness and social isolation are associated with poorer health and behaviours.^23–25^ While the negative impact of unemployment on social engagement in those of working age is well understood,^1^ research specifically focusing on older people or considering other exits from the workplace, such as sickness/disability or retirement, is limited, being largely cross-sectional or restricted to very short follow-up periods. Evidence from the current study, based on 15 years of follow-up, suggests that leaving employment in older age, for any reason, has a substantial negative impact on social engagement and self-esteem. The greater impact of sickness/disability and unemployment is, perhaps, unsurprising, but these results, along with those for retirees and older (female) home makers, who may have left the workplace to care for other family members, are significant causes for concern.

Previous studies of employed individuals have highlighted the negative health consequences of stress and lack of control and autonomy in the workplace,^27 28 40^ while retirement, particularly from jobs with poor working conditions, may lead to health improvements.^13–15^ Our results support the beneficial effects of retirement in this regard, with respondents in retired waves similar or less likely than in employed waves to agree that their status was stressful or resulted in a lack of control/autonomy. However, results for individuals leaving the workplace through other routes were less positive, with older respondents in unemployed or sick/disabled waves more likely than in employed (or retired) waves to agree that their status prevented them from feeling in control and caused them a lot of worry. These results suggest that, while some workplace exits offer relief from employment-related stress, this is not the case for all routes and, in particular, enforced non-employment via redundancy or ill health may lead to an increase in worry and loss of control.

Several of our results are contrary to popular portrayals of older non-employed people. Individuals of all ages who are not working due to sickness/disability or unemployment are often identified in the media as ‘scroungers’ or ‘happy slackers’.^41 42^ However, this view is not supported by our results, which indicate that older adults who are not employed for these reasons often have very negative feelings towards their status across all of our themes. In addition to the multiple risk factors for poor health explored here, there are additional specific risks as a result of the stigma of these employment states,^30 31^ particularly in the long term, and non-employed individuals represent high-risk groups for targeted intervention. Also of note are results for retirees, who were generally negative about social engagement and self-esteem, contradicting perceptions of retirement as a time of increased activity and socialising for all, particularly given that respondents were aged 70 years or less and therefore pre-dating the majority of physical limitations associated with extreme old age. In light of increasing longevity worldwide, there is a rapidly expanding interest in the determinants of successful ageing, including continued social and productive engagement.^9 10^ Inevitably, some individuals remain in employment longer than they wish, most commonly due to financial constraints, but it is more common for older people to retire at a younger age than they would like^16^ and our results suggest that there are substantial negative implications, consistent with previous work highlighting the psychosocial advantages of employment.^21^ Exits from the workplace are often permanent in older individuals and re-employment may not be realistic or practical, but interventions targeting mechanisms such as social engagement and self-esteem (eg, programmes promoting volunteering and community involvement or widening access to public transport) provide potential pathways towards reducing the negative impact of non-employment on health in older adults.

### Conclusion

There are many individual and societal benefits to extending working lives and the success of future pension systems may be highly reliant on increases in the older workforce and retirement ages. However, recent trends have been towards earlier exits from the workplace, not always through choice. Compared with those still working, and in contrast to many popular perceptions, older people who are not in employment, particularly those out of work due to sickness/disability or unemployment, are more likely to feel that their employment status results in poorer social and mental engagement, lower self-esteem, and greater stress, worry and lack of control. These individuals represent a high-risk group and, while re-employment may not always be possible, these mechanisms offer an important opportunity to improve outcomes and promote successful ageing.
