# Supplementary material for: Leaving the labour market later in life: how does it impact on mechanisms for health?
Source: Occup Environ Med. 2017 Aug 21;74(12):877–86. doi: 10.1136/oemed-2016-104258 (PMC5740548; doi:10.1136/oemed-2016-104258)

Figure S1: Between person differences in feelings about employment status (functioning and mental engagement) comparing non-employed versus employed waves

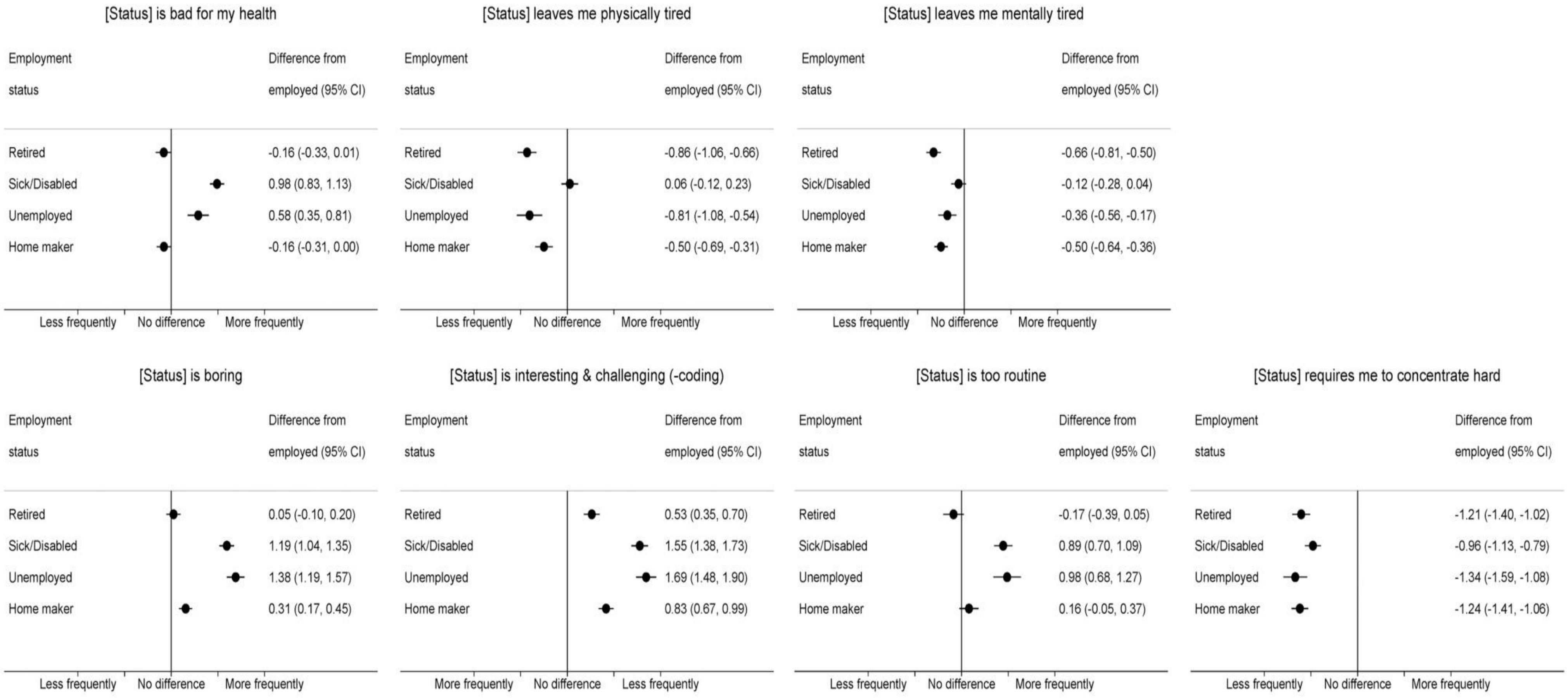

Figure S2: Between person differences in feelings about employment status (social engagement and self-esteem) comparing non-employed versus employed waves

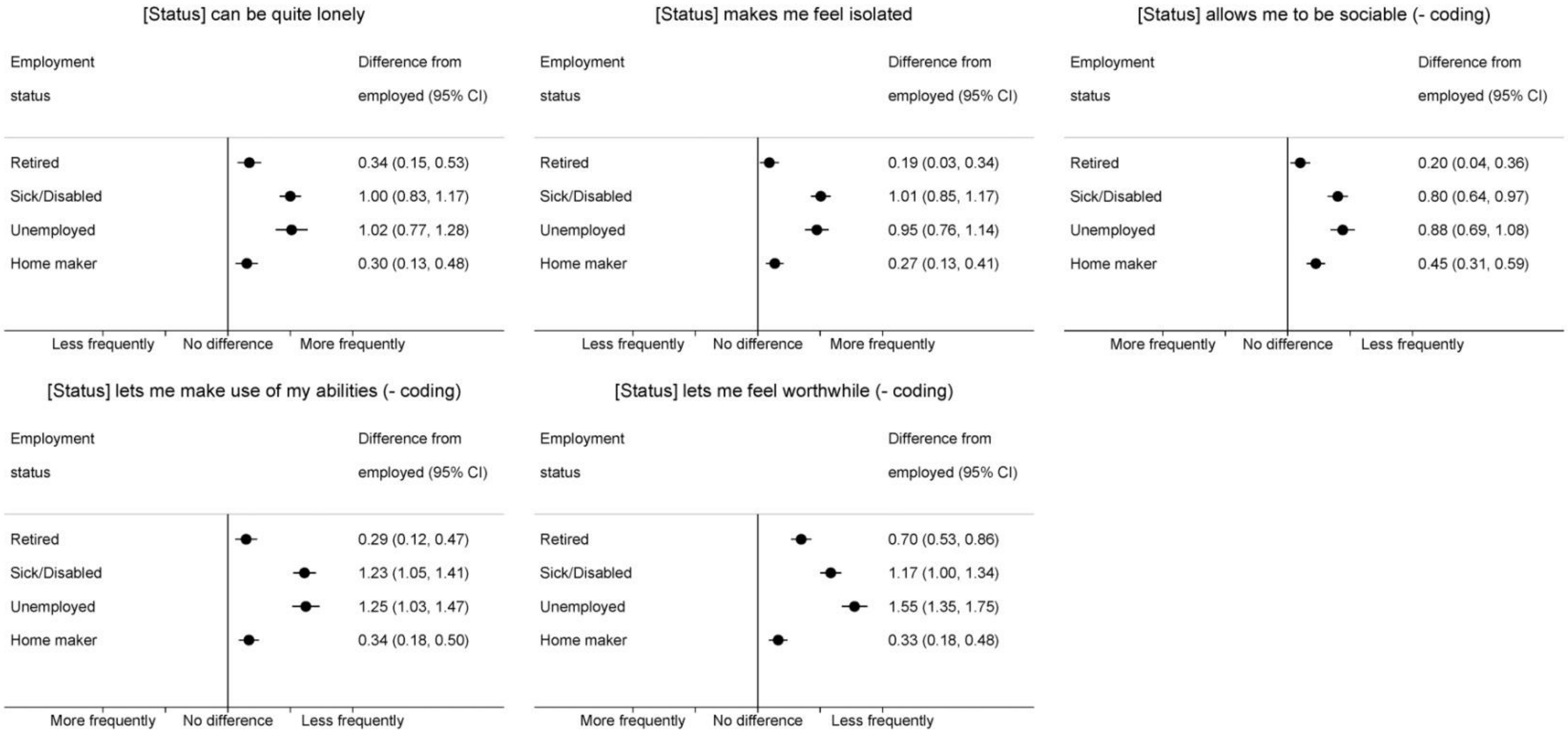

Figure S3: Between person differences in feelings about employment status (stress and control and autonomy) comparing non-employed versus employed waves

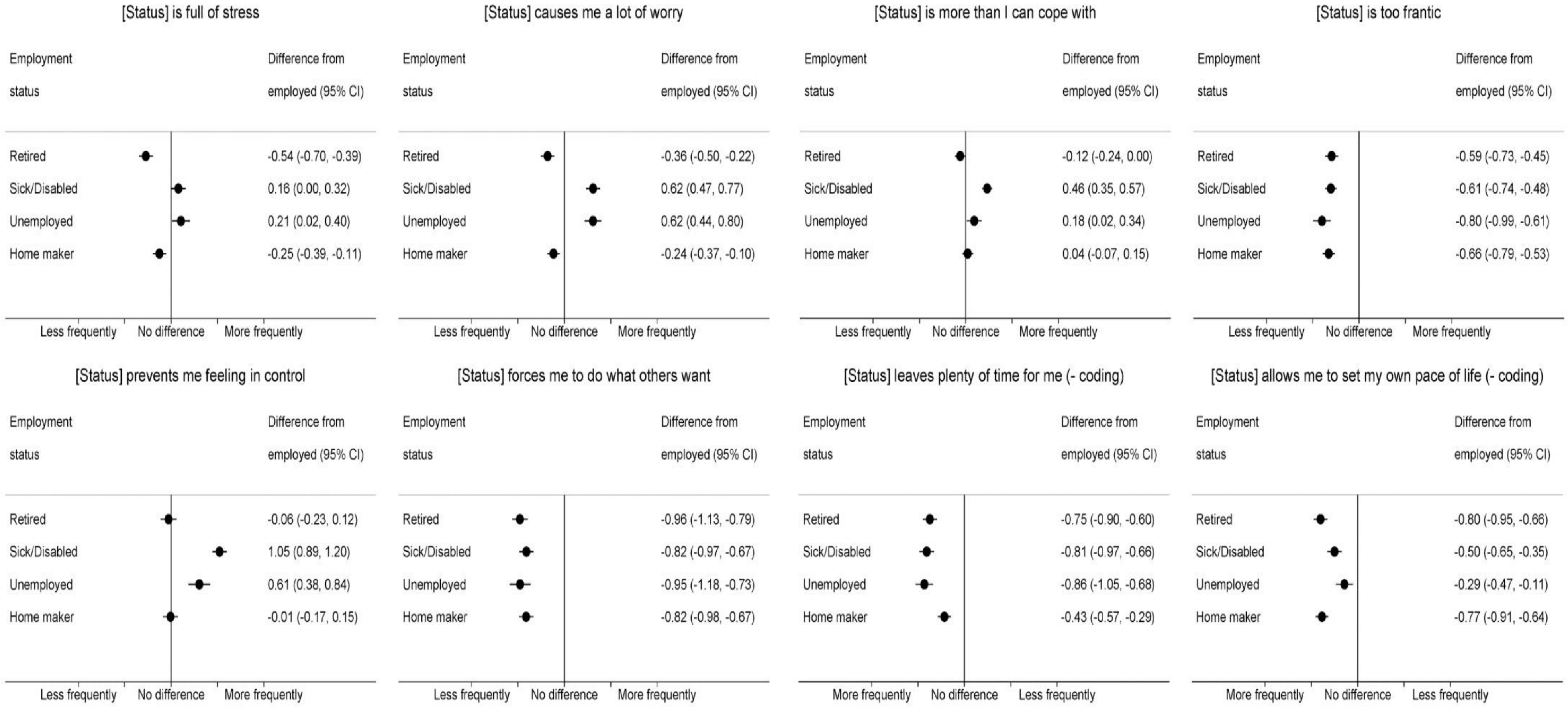

Supplement: Supplementary material 1 [file oemed-2016-104258supp001.pdf]
